# CBCT for accurate diagnosis of oral lesions: Comparison with biopsy and imaging

**DOI:** 10.6026/973206300213391

**Published:** 2025-09-30

**Authors:** Mansha Zarabi, Gagandeep Gupta, Vikram Bali, Rajneesh Parimoo, Tanya Goyal, Shivangi Gupta

**Affiliations:** 1 Department of Periodontics and Oral Implantology, Desh Bhagat Dental College and Hospital, Mandi Gobindgarh, Punjab, India; 2 Department of Paediatric and Preventive Dentistry, Desh Bhagat Dental College and Hospital, Mandi Gobindgarh, Punjab, India

**Keywords:** Biopsy, CBCT, Diagnostic accuracy, Oral lesions, Sensitivity

## Abstract

Diagnosing oral lesions accurately is challenging because conventional methods like biopsy, while reliable, are invasive, time-consuming, and may cause discomfort or complications. Therefore, it is of interest to compare the results of cone-beam computed tomography (CBCT) with biopsy results to assess the accuracy of CBCT in locating oral lesions. This study consisted of two groups of participants, totaling 108 individuals. Group A consisted of participants who underwent biopsy and the other group underwent imaging using CBCT. Data shows that CBCT was better at detecting abnormalities. Thus, we show that CBCT is a safe and reliable approach for checking bone involvement, malignancies, and soft tissue expansion.

## Background:

It is very important to diagnose and treat oral lesions right away and in the right way to avoid having serious diseases like cancer or conditions that can lead to cancer [1]. It can be hard to tell what's wrong when someone has oral disorders such as cysts, tumors or swelling. A normal tissue biopsy is one of the most reliable methods for identifying oral lesions [2]. A dentist takes a small piece of tissue from the affected area and examines it under a microscope to determine whether any cells that shouldn't be there are present [3]. A biopsy gives us clear information, but it is an invasive process that may require anesthesia and could cause discomfort, bleeding and infections. It will take longer than expected to receive the biopsy findings, as the samples must be sent to a laboratory where a microscope can be used to examine them [4]. Cone Beam Computed Tomography (CBCT) utilizes X-rays to examine oral lesions without requiring direct contact with the tissues. CBCT generates highly detailed three-dimensional images of the mouth and jaw, which assist dentists in identifying issues. In contrast, 2D imaging only shows pictures in two dimensions [5].

CBCT takes three-dimensional photos that show the size of the lesion and how hard and soft tissue differs. This is why this method is currently quite helpful for identifying disorders that affect the teeth, jawbones and surrounding soft tissues. Many dentists utilize CBCT imaging because it is non-invasive and can detect structural changes more quickly than regular biopsies [6]. More and more dentists are using CBCT to make diagnoses, but it's still important to compare its accuracy to that of regular X-rays and biopsies. Dentists use X-rays frequently, but they often don't provide the clear information necessary to diagnose a patient's oral health issues [7]. CBCT provides more detailed information, enabling the localization of bone lesions, revealing their proximity to adjacent bones and detecting changes that standard biopsies may miss [8]. Therefore, it is of interest to describe the potential of CBCT as a safe and non-invasive method for identifying oral health issues, offering an alternative to traditional diagnostic approaches.

## Methodology:

The goal of this study was to determine the effectiveness of CBCT and regular biopsies in diagnosing oral lesions. The study was done by the Department of Periodontics and Oral Implantology at Desh Bhagat Dental College and Hospital. The study consisted of 108 participants, divided into two groups of 54. Participants in Group A had their lesions scanned with CBCT, whereas those in Group B had their lesions biopsied. To be chosen, patients had to be at least 18 years old and have lesions in their mouths that required examination. People who couldn't receive CBCT imaging due to cost issues, those who had already undergone a biopsy for the same lesion and individuals with systemic conditions that could complicate lesion detection were excluded. In Group A, CBCT scans were performed, displaying the oral lesions in three dimensions. These scans showed how the lesions progressed through the soft tissue and affected the bone. Radiologists who had already examined this area used these pictures to determine the location, size, and appearance of the lesions.

We used a microscope to examine samples of the lesions in Group B to ensure the accuracy of the diagnosis. We examined the effectiveness of CBCT and biopsy in conjunction, assessing their accuracy, sensitivity and ability to predict outcomes. We used statistics to determine the accuracy, sensitivity and specificity of each test when all were performed simultaneously. After that, we analyzed the data using statistical tests, such as the chi-square test and the t-test. The institutional review board determined that the study was ethical and that all participants understood the nature of their involvement. This ensured their information remained private and safe throughout the trial. The goal of this research was to demonstrate that CBCT can detect oral lesions without incising into the skin and that it is as accurate as a regular biopsy.

## Results:

Table 1 (see PDF) shows that Group A (CBCT) consisted of 28 males and 26 females, while Group B (Biopsy) consisted of 29 males and 25 females, resulting in an overall sample of 57 males and 51 females. In the study, the oral lesions were classified into two main categories: benign and malignant lesions. The distribution of these lesion types across both diagnostic methods (CBCT and biopsy) was recorded for comparison. The following table shows the distribution of lesion types in Group A (CBCT) and Group B (Biopsy). Table 2 (see PDF) shows that benign lesions were the most common in both groups, with Group A (CBCT) showing a slightly higher proportion of benign lesions (70%) compared to Group B (Biopsy) (67%). Malignant lesions were less frequent, occurring in 30% of cases in Group A and 33% in Group B. The overall distribution of lesions was fairly consistent between both groups. CBCT was 95% sensitive, meaning it correctly identified 95% of the lesions confirmed by biopsy. The biopsy was 90% accurate, meaning it correctly identified 90% of the lesions. In other words, CBCT was slightly better than biopsy in detecting bone lesions, malignancies, and soft tissue growths. CBCT was 91% specific, meaning it correctly identified 91% of individuals who did not have lesions. Biopsy, on the other hand, was 90% specific, meaning it identified 90% of cases where there was no lesion. CBCT was a little more specific because it could provide more thorough information about the structure. When CBCT found a lesion, it was correct 93% of the time, which is a very high positive predictive value (PPV). The biopsy had a positive predictive value (PPV) of 94%, meaning that it provided a correct positive diagnosis. There was little difference in PPV between the two techniques, indicating that both performed quite well in confirming lesions. The negative predictive value (NPV) of CBCT was 97%, suggesting that it correctly identified the absence of a lesion in 97% of cases. The NPV for biopsy, on the other hand, was 95%. CBCT had a slightly higher NPV, indicating it was better at ruling out lesions when there were no concerns.

CBCT and biopsy agreed on the diagnosis 92% of the time. This means that in 92% of situations, both approaches decided on whether or not there was a lesion. However, there were times when biopsies revealed tiny details that CBCT couldn't observe, especially in tumors that were too small or involved intricate cellular structures. We used a Chi-Square Test to determine how benign and malignant lesions were distributed in Group A (CBCT) and Group B (Biopsy). The p-value of 0.12 indicated that there was no significant difference between the two types of lesions identified by CBCT and biopsy (p > 0.05). This means that both methods find lesions in the same way. We also utilized the McNemar Test to compare the two methods, CBCT and biopsy, to evaluate which one was superior at discovering lesions in the mouth. The results showed that the two approaches were not significantly different in terms of sensitivity, specificity, PPV and NPV (p > 0.05). This means that both CBCT and biopsy are equally good in finding lesions in the mouth.

## Discussion:

CBCT is a less invasive approach to biopsy than other methods. According to a study by Kanneppady *et al.* (2025) [9], CBCT is superior to conventional radiography in detecting bone lesions and soft tissue expansions due to its greater specificity and sensitivity. He stated that CBCT produced superior three-dimensional images, which made it easier to detect lesions early and more accurately than standard X-rays. This study reveals that CBCT can find bone lesions. According to Straub *et al.* (2024) [10], CBCT was better at diagnosing difficulties with oral lesions. This aligns with our study; biopsy is a reliable way to determine malignancies, but CBCT provides more information regarding diagnosis, especially when it comes to identifying issues that regular two-dimensional X-rays might miss. According to a 2017 study by Shukla *et al.* [11], biopsy is particularly critical for making an accurate diagnosis. However, CBCT is an even more effective way to diagnose because it can display the size and position of the lesion in three dimensions, which helps clinicians determine the best course of treatment. This aligns with our study, which found abnormalities at the microscopic level that could only be seen with a biopsy, but not with CBCT. Although CBCT has some limitations, it remains a much better technique than biopsy.

## Conclusion:

Both CBCT and biopsy exhibited similar diagnostic accuracy for oral lesions, with CBCT providing marginally improved sensitivity and negative predictive value is shown. CBCT offers a non-invasive, dependable alternative to biopsy, especially for identifying bone involvement and soft tissue extension. Thus, we show that CBCT can serve as an effective adjunctive tool in the diagnosis of oral lesions.
